# 667. Next Generation Sequencing of Microbial Cell Free DNA in the Diagnosis and Treatment of Infectious Disease in Children: When Does the Result Justify the Cost?

**DOI:** 10.1093/ofid/ofab466.864

**Published:** 2021-12-04

**Authors:** Rachel Downey Quick, Kelli A Martinez, Susan M Russo, Sarah E McGwier, Rachel A Quirt, Sarmistha Bhaduri Hauger, Mariosl Fernandez, Donald Murphey

**Affiliations:** 1 Dell Children's Medical Group, Austin, TX; 2 Dell Medical School at the University of Texas at Austin, Austin, TX; 3 Dell Children’s Medical Group, Autsin, TX; 4 Dell Children's Medical Center, University of Texas at Austin Dell Medical School, Austin, TX; 5 Dell Children's Medical Center; University of Texas at Austin Dell Medical School, Austin, TX

## Abstract

**Background:**

Pathogen testing using next-generation sequencing of microbial cell-free DNA (NGS cfDNA) is a promising diagnostic tool to identify pathogens that might not be detected using conventional lab evaluation. Considering the cost of this test, it is important to determine when it is most useful to the plan of care (POC).

Figure 1. Unit of admission among cases

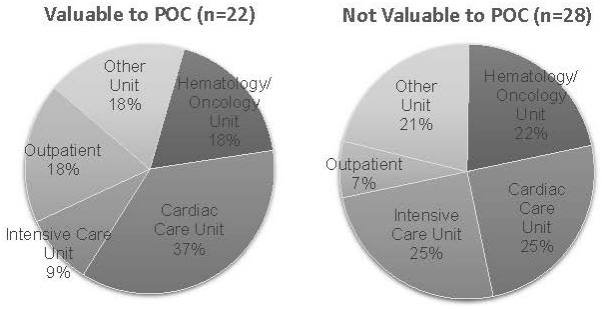

Figure 2. Patient characteristics in cases determined to be valuable and not valuable to the plan of care (POC)

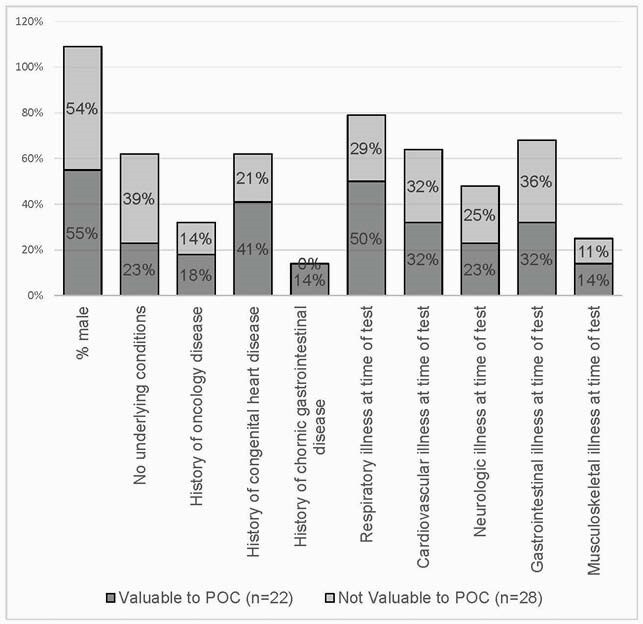

**Methods:**

In this retrospective study, we collected data from the medical charts of 50 consecutive NGS cfDNA tests in a free-standing children’s hospital. We evaluated patients for demographics, underlying conditions, diagnosis at time of testing, conventional laboratory testing and timing, medical treatment, and NGS cfDNA test results for clinical relevance or false negative results compared to conventional testing. The primary goal was to identify patients for whom the NGS cfDNA testing affected the POC. Charts were reviewed, and determinations regarding whether the result influenced the POC were confirmed by a provider.

**Results:**

We were unable to differentiate patients with clinically valuable NGS cfDNA results (Fig 1 & 2). Among those with NGS cfDNA results valuable to the POC (n=22), both negative and positive testing guided POC (13 valuable negative vs. 9 diagnostic cases). In the total sample, 5 cases (10%) had a clinically relevant pathogen identified through conventional testing, but not through NGS cfDNA and 2 cases had antimicrobial resistance on culture, which is not detected by NGS cfDNA.

**Conclusion:**

While we did not find a specific clinical profile for NGS cfDNA use, positive results were essential to the diagnosis in 18% of cases with otherwise negative laboratory evaluation for the pathogen identified in NGS cfDNA. Negative tests affected the POC in 26% of cases by avoiding unnecessary antimicrobials in high risk immunocompromised patients and patients that presented with low-risk of infection, but unclear disease process.

Caution must be exercised with reliance on this test with respect to antimicrobial resistance and risk of false negative results.

**Disclosures:**

**All Authors**: No reported disclosures

